# Supplementary material for: The Impact of Practicing Open- vs. Closed-Skill Sports on Executive Functions—A Meta-Analytic and Systematic Review with a Focus on Characteristics of Sports
Source: Brain Sci. 2022 Aug 12;12(8):1071. doi: 10.3390/brainsci12081071 (PMC9406193; doi:10.3390/brainsci12081071)
Supplement: Supplementary file 1 [file brainsci-12-01071-s001.zip › brainsci-1848427-supplementary.pdf]

Table S1.
Characteristics of the cross-sectional studies eligible for qualitative and quantitative analysis (alphabetical order)

**Study (Author, Year)**	**Sample Size** Total OSE CSE Control **(Female/male)**	**Sample Age in Years** OSE CSE, Control **(M/SD)** **(Age Category)**	**EFs**	**EF-measure**	**OSE**	**CSE**	**Level of performance / Experience**	**Outcome** Regarding the difference between OSE vs. CSE
**Ballester et al. [30]**	*N* = 66<br>22 (8/14)<br>22 (10/12)<br>22 (--/--)	24.6 (0.9)<br>22.5 (0.9)<br>22.3 (0.6)<br>(Adults)	IC	Oddball Task	Basketball, Football, Martial arts, Tennis, Volleyball	Athletics, Cycling, Swimming Triathlon	No differences in years of training (p = .285) but in hours per week (p = .019, d = .75, OSE < CSE)	- Sig. effect of type of sport on inhibition control (OSE > CSE)
**Chang et al. [37]**	*N* = 60<br>20 (5/15)<br>20 (6/14)<br>20 (7/13)	21.15 (1.2)<br>21.20 (1.8)<br>21.60 (1.4)<br>(Adults)	IC, CF	Stroop Task, WCST	Wushu training	Marathon running	No differences in training hours and training years between OSE and CSE, national level	- No sig. effects between groups in Stroop or WCST performance (OSE > CSE)
**Chen et al. [53]**	*N* = 60<br>23 (4/19)<br>24 (15/9)<br>23 (11/12)	57.17 (3.2)<br>59.08 (7.2)<br>58.91 (4.8)<br>(Adults)	WM	Spatial Working Memory Task	Badminton, Basketball, Table tennis, Tennis	Cycling, Running, Swimming	Sig. difference in IPAQ (F[2,67] = 8.30, p < .001, mean IPAQ scores: CSE > OSE > control)	- Sig. main effects of group (F[2,67] = 5.73, p < .01, η_p^2 = 0.15) for accuracy but no difference between OSE and CSE (*ps* < .05)<br>- No sig. main effects for RT<br>for group (F[2,67] = 0.69, p > 0.05, η_p^2 = 0.02)
**Chueh et al. [38]**	*N* = 48<br>16 (7/9)<br>16 (7/9)<br>16 (7/9)	20.00 (1.2)<br>21.10 (2.3)<br>20.70 (1.1)<br>(Adults)	WM	Non-delayed and delayed match-to-sample task	Badminton, Table tennis	Running, Swimming, Triathlon	Differences in years of training between OSE (10.8 ± 2.2) and CSE (9.7 ± 3.2)	- No main effects of group for accuracy results (F[2,45] = 0.025, p = .096)<br>- Sig. effects of group for RT results (F[2,45] = 5.11, p < .05, η^2 = .185) but no

								sig. effects between OSE and CSE groups (OSE: 632.52 ms; CSE 655.03 ms < Con: 736.66 ms)
**Dai et al. [39]**	*N* = 48<br>16 (7/9)<br>16 (10/6)<br>16 (14/2)	 69.00 (3.6)<br>69.90 (3.6)<br>67.30 (3.0)<br>(Older Adults)	CF	Task-switching paradigm	Table tennis, Tennis	Running, Swimming	Differences in years of training between OSE (13.0 ± 5.7) and CSE (11.1 ± 5.4)	- Sig. differences for RT compared with control group (*ts* (30) > 2.46, *p* < .02) for homogeneous condition, no sig. differences between OSE and CSE<br>- Sig. main effect for exercise mode for global switch costs ($F[2,45] = 3.52$, $p = .03$, $\eta^2 = .14$) with differences between OSE and CSE but not for local switch cost ($F[2,45] = .20$, $p = .82$)
**DeWaelle et al. [51]**	*N* = 170<br>86 (86/--)<br>25 (25/--)<br>59 (59/--)	 10.20 (1.0)<br>10.30 (1.1)<br>10.40 (1.1)<br>(Children)	IC, WM	Spatial Span Task, Token Search, Monkey Ladder, Double Trouble Task,<br>Sustained Attention to Response Task	Basketball, Hockey, Korfball, Soccer, Volleyball	Athletics, Cycling, Swimming,	At least 2 h/week, no differences between OSE and CSE	- Sig. main effect for group ($F[2,166] = 5.143$, $p = .007$, $\eta_p^2 = .058$) with superior executive functions of OSE compared with CSE and control group
**Gökce et al. [55]**	*N* = 54<br>18 (9/9)<br>18 (9/9)<br>18 (11/7)	 20.44 (1.9)<br>21.00 (2.0)<br>22.33 (1.9)<br>(Adults)	WM	Corsi's Block Tapping Test	Fencing	Swimming	No sig. differences in training history between<br>OSE group<br>(8.27 ± 2.13 years) and CSE group (8.22 ± 2.66 years)	- Sig. differences for right- and left-handed CBT, left hemispace accuracy was significantly different among the groups (right: $H(2) = 15.336$, $p < .001$, $\eta^2 = .28$; left: $H(2) = 12.318$, p = .002, $\eta 2 = .23$)<br>- OSE higher than CSE($p = 0.034$, $r = -0.41$) and controls ($p < .001$, $r = -0.65$)

								- Both OSE (p = .017, r = -.46) and CSE (p = .003, r = -0.53) higher accuracy scores than controls
**Guo et al. [40]**	N = 111<br>36 (19/17)<br>38 (23/15)<br>37 (21/16)	67.6 (5.9)<br>66.7 (5.8)<br>66.9 (5.9)<br>(Older Adults)	WM	Visuospatial WM, short-term memory & mental rotation task	Badminton Table Tennis	Running, Swimming	No differences in training hours and training years between OSE and CSE	- Sig. effect of group on short-term memory task accuracy ($F(2, 99)$ = 3.68, p = .02), but no differences between OSE and CSE<br>- No sig. differences in reaction time among the groups ($F(2, 99)$ = 0.54 p = .58)
**Holfelder et al. [56]**	N = 86<br>46 (23/23)<br>40 (20/20)	13.85 (0.7)<br>14.05 (0.8)<br>(Preadolescents)	IC, WM, CF	Flanker Task,<br>n-Back Task,<br>Trail Making Task	Handball	Athletics	Amateur and elite athletes of OSE and CSE	- No effect of type of sport on working memory, inhibitory control or cognitive flexibility<br>- specific Trail Walking Task sig. group effect (p < .01; no standard EF-measure)
**Huang et al. [41]**	N = 60<br>20 (9/11)<br>20 (11/9)<br>20 /14/6)	69.4 (3.0)<br>70.55 (2.6)<br>68.3 (2.3)<br>(Older Adults)	IC	Ericsen Flanker Task	Table Tennis, Tennis, Badminton	Running, Swimming	Differences in participation regularity (OSE > CSE), exercise duration (OSE > CSE) and weekly frequency (OSE < CSE)	- Sig. effect of goup on inhibition control ($F(2,57)$ = 3.33, p = .043)<br>- OSE (441.94 ms) and CSE athletes (446.45 ms) exhibiting shorter RTs than controls (476.11 ms)
**Jacobson & Matteaus [8]**	N = 54<br>22 (8/14)<br>17 (14/3)<br>15 (9/6)	20.05 (1.2)<br>20.18 (1.5)<br>20.20 (1.3)<br>(Adults)	IC	D-KEFS Tower Test & Color-Word interference Test	Soccer, Tennis	Running, Swimming	Differences between high-skilled and recreational Athletes	- Sig. effect of group on inhibition control ($t(52)$ = 2.94, p = .003, r = .37); OSE > CSE
**Koch & Krenn [27]**	N = 75<br>44(18/26)<br>31(12/19)<br>-	22.89 (4.2)<br>23.23 (4.7)<br>(Adults)	WM, IC, CF	Design Fluency Test, Trail Making Task, Flanker Task, 2-back Task	American football, Basketball, Canoe slalom, Handball,	Archery, Athletics, Cross-country skiing,	Elite athletes	- Sig. main effect of group on cognitive flexibility ($F(3, 70)$ = 2.55, p = .03) and working memory ($F(3, 70)$ = 3.05, p = .02); OSE > CSE

					Olympic sailing	Marathon, Shooting, Swimming, Track-bike, Triathlon		
**Li et al. [57]**	*N* = 75<br>25 (10/15)<br>25 (17/8)<br>25 (21/4)	69.04 (3.4)<br>69.80 (3.1)<br>67.80 (2.9)<br>(Older Adults)	IC, CF	Stroop Task, Task-switching	Table tennis, tennis	Running, Brisk walking	No differences in physical activity in exercise groups	- Sig. effect of group on inhibition control (*F(2,66)* = 4.60, *p* = .013) and global switch costs in task switching (*F(2,24)* = 7.03, *p* = .004)<br>- No reported difference between OSE and CSE
**Nakamoto & Mori [50]**	*N = 18*<br>9 (--/9)<br>9 (--/9)	--	IC	Go/NoGo Task	Baseball	Athletics, Gymnastics	OSE group spent 25 h/week for baseball training, no data for CSE group	- No significant group difference (*t*(16)=1.65, p < .05) for simple reaction time<br>- Sig. group differences for Spatial-BB, Spatial-Mix and color condition(*p* < .01)
**Tsai & Wang [22]**	*N* = 64<br>21 (7/14)<br>22 (8/14)<br>21 (8/13)	65.35 (4.2)<br>66.03 (4.1)<br>63.94 (3.4)<br>(Older Adults)	CF	Task switching	Badminton, Tabletennis	Running, Swimming	No differences in training<br>(3 x 30 min/week) and training years (2 years)	- OSE responded faster in switch condition than CSE<br>- sig. main effect of group on specific RT switch costs (*F*[2,61] = 7.30, *p* = .001)<br>-
**Wang & Guo [42]**	*N* = 259<br>85 (45/40)<br>87 (49/38)<br>87 (46/41)	66.8 (5.5)<br>65.5 (5.8)<br>65.9 (6.3)<br>(Older Adults)	IC	Attention network test (ANT)	Badminton, Tennis	Running, Swimming	No differences in training<br>(3 x 30 min/week) and training years (1 year)	- OSE significantly higher executive network efficiency than CSE (*p* < .01)<br>- No differences for alerting and orienting networks (*p* > .05) and for proportion scores of alerting and orienting networks
**Wang et al. [26]**	*N* = 60<br>20 (-/20)<br>20 (-/20)<br>20 (-/20)	20.70 (2.4)<br>19.31 (0.8)<br>20.40 (2.1)	IC	Stop-signal task	Tennis	Swimming	No sig. differences [t(39) =.89, p= .381, d= 0.28] in years of training between OSE	- OSE had shorter stop-signal reaction times than CSE *(t*(39) = 3.76, *p* = .001)

		(Adolescents/Adults)					(5.50 ± 2.80) and CSE (4.85 ± 1.64)	- no differences on RTs between OSE and CSE<br>- No significant differences among groups on inhibition function ($F(2, 57) = .02$, $p = .981$, $\eta_p^2 = .00$)
**Yamashiro et al. [52]**	$N = 24$<br>12 (-/12)<br>12 (-/12)<br>--	 21.2 (0.8)<br>22.7 (3.4)<br>--<br>(Adults)	IC	Go/Nogo paradigm	Basketball	Athletics, Swimming	No differences	- Go/Nogo RT shorter for OSE than CSE ($p = .07$).
**Yu et al. [43]**	$N = 54$<br>18 (8/10)<br>18 (7/11)<br>18 (9/9)	 21.1 (2.2)<br>21.1 (2.2)<br>21.8 (2.1)<br>(Adults)	CF	Cued task-switching paradigm task	Badminton	Athletics	No differences in years of training levels of skill competencies between OSE and CSE	- sig. fewer switch costs in OSE than CSE in 100% validity condition ($p = .023$)<br>- No significant differences in switch costs between OSE and CSE ($p = .473$)

Note: EFs = Executive Functions, OSE = Open-Skill-Exercise, CSE = Closed-Skill-Exercise, WM = Working-Memory, IC = Inhibition Control, CF = Cognitive Flexibility, RT = reaction time, ACC = accuracy
